# Alternatively activated macrophages are associated with the α2AP production that occurs with the development of dermal fibrosis: The role of alternatively activated macrophages on the development of fibrosis

**DOI:** 10.1186/s13075-020-02159-2

**Published:** 2020-04-10

**Authors:** Yosuke Kanno, En Shu, Hirofumi Niwa, Hiroyuki Kanoh, Mariko Seishima

**Affiliations:** 1 Department of Clinical Pathological Biochemistry, Faculty of Pharmaceutical Science, Doshisha Women’s College of Liberal Arts, 97-1 Kodo Kyo-tanabe, Kyoto, 610-0395 Japan; 2 Department of Dermatology, Graduate School of Medicine, Gifu University, 1-1 Yanagido, Gifu, 501-1194 Japan

**Keywords:** Fibrosis, α2AP, HMGB1, Alternatively activated macrophages, IL-4Rα

## Abstract

**Background:**

Fibrotic diseases are characterized by tissue overgrowth, hardening, and/or scarring because of the excessive production, deposition, and contraction of the extracellular matrix (ECM). However, the detailed mechanisms underlying these disorders remain unclear. It was recently reported that α2-antiplasmin (α2AP) is elevated in fibrotic tissue and that it is associated with the development of fibrosis. In the present study, we examined the mechanism underlying the production of α2AP on the development of fibrosis.

**Methods:**

To clarify the mechanism underlying the production of α2AP on the development of fibrosis, we focused on high-mobility group box 1 (HMGB1), which is associated with the development of fibrosis. The mouse model of bleomycin-induced fibrosis was used to evaluate the production of α2AP on the development of fibrosis.

**Results:**

We found that HMGB1 induced the production of α2AP through receptor for advanced glycation end products (RAGE) in fibroblasts. Next, we showed that macrophage reduction by a macrophage-depleting agent, clodronate, attenuated the progression of fibrosis and the production of α2AP and HMGB1 in the bleomycin-induced mice. We also showed that IL-4-stimulated alternatively activated macrophages induced the production of HMGB1, that IL-4-stimulated alternatively activated macrophage conditioned media (CM) induced pro-fibrotic changes and α2AP production, and that the inhibition of HMGB1 and RAGE attenuated these effects in fibroblasts. Furthermore, the blockade of IL-4 signaling by IL-4Rα neutralizing antibodies attenuated the progression of fibrosis and the production of α2AP and HMGB1 in the bleomycin-induced mice.

**Conclusion:**

These findings suggest that alternatively activated macrophage-derived HMGB1 induced the production of α2AP through RAGE and that these effects are associated with the development of fibrosis. Our findings may provide a clinical strategy for managing fibrotic disorders.

## Introduction

Fibrotic diseases are characterized by tissue overgrowth, hardening, and/or scarring due to excessive production, deposition, and contraction of the extracellular matrix (ECM). This process usually occurs over many months and years, and can lead to organ dysfunction or death. It has been reported that immune cells, such as alternatively activated macrophages, T cells, and B cells, have been found in the tissues with fibrotic disease and that immune cells induce the production of pro-fibrotic factors and ECM [1–5]. Although abnormality of the innate and adaptive immune systems induced by the increase in these immune cell numbers may contribute to the development of fibrosis, the detailed mechanism remains unclear.

Alpha2-antiplasmin (α2AP) is known to be the principal inhibitor of plasmin, resulting in the formation of a stable inactive complex, plasmin-α2AP (PAP), and inhibits fibrinolysis and ECM degradation [6–8]. α2AP is synthesized in various tissues; has various functions, such as cytokine production, cell growth, and cell differentiation; and regulates angiogenesis, inflammatory response, immune modulation, tissue repair, bone formation, and brain functions [9–18]. Many studies have reported that the levels of PAP in plasma are elevated in fibrotic diseases which include systemic sclerosis (SSc), diabetic nephropathy, liver cirrhosis, and rheumatoid arthritis [19–22]. Recently, we showed that the expression of α2AP is elevated in fibrotic tissue [11, 23–25], and α2AP induces the transforming growth factor-β (TGF-β) production through adipose triglyceride lipase (ATGL), which has been described as a member of the calcium-independent phospholipase A_2_ (iPLA_2_)/nutrin/patatin-like phospholipase domain-contain 2 (PNPLA2) family, and is associated with pro-fibrotic effects, such as cytokine production, myofibroblast differentiation, and ECM production [11, 26]. α2AP also inhibits the activity of plasmin, which can directly and indirectly degrade a number of matrix proteins by activating latent metalloproteinases (MMPs) [8] and activate hepatocyte growth factor (HGF) [27, 28]. Furthermore, the blockade of α2AP or α2AP deficiency attenuates the development of fibrosis in humans and mice [11, 25, 29], and the increase of α2AP expression may play a critical role in the fibrotic disease severity. However, the mechanism underlying the production of α2AP that occurs with the process of fibrosis progression remains unclear.

High-mobility group box 1 (HMGB1) is a multifunctional protein that exerts pro-inflammatory activity by mainly binding to receptor for advanced glycation end products (RAGE). It has been reported that HMGB1 is released from immune cells, and contributes to inflammation, immune responses, myofibroblast differentiation, ECM production, and fibrosis progression [30–34], and the HMGB1 inhibitor, glycyrrhizin, attenuates the development of fibrosis in the belomycin-treated mice [35].

In the present study, we examined the mechanism underlying the production of α2AP in the development of fibrosis and showed that alternatively activated macrophage-derived HMGB1 induced α2AP production through RAGE in fibroblasts and that the blockade of IL-4 signaling by IL-4Rα neutralization suppressed these effects, resulting in the improvement of fibrotic disorder.

## Methods

### Mice experiment

We induced dermal fibrosis in mice by bleomycin as previously described [29]. Bleomycin was dissolved in saline at 1 mg/ml. The saline or bleomycin was administered subcutaneously into the shaved back of the mice (male 8-week-old SCID mice). The administration of saline or bleomycin in the same site was carried out daily for up to 2 weeks. In other experiments, the saline, bleomycin, saline plus clodronate (FormuMax Scientific, CA, USA), or bleomycin plus clodronate were similarly administered to the mice (male 8-week-old C57BL/6 J mice). The administration of saline or bleomycin in the same site was carried out daily for up to 2 weeks, and the administration of clodronate was carried out intraperitoneally every 3 days for up to 2 weeks. In addition, the saline or bleomycin was similarly administered to the mice (male 8-week-old C57BL/6 J mice). The administration of saline or bleomycin in the same site was carried out daily for up to 2 weeks, and the administration of control IgG (2 mg/kg) or anti-IL-4Rα antibodies (2 mg/kg) (BD Biosciences, NJ, USA) was administered intraperitoneally every week for up to 2 weeks.

### Cell culture

The dermal fibroblasts were obtained from mice as previously described [9]. The dermal fibroblasts or RAW 264.7 macrophages were seeded into the 35-mm-diameter dishes and maintained in 2 ml Dulbecco’s modified Eagle’s medium (DMEM) containing 10% fetal calf serum (FCS) at 37 °C in a humidified atmosphere with 5% CO_2_/95% air. After 6 days, the medium was replaced with serum-free DMEM. Then, the cells were used for experiments. HMGB1 was obtained from Assaypro (MO, USA). Glycyrrhizin was obtained from Nacalai Tesque (Kyoto, Japan). FPS-ZM1 was obtained from Focus Biomolecules (PA, USA).

### Western blot analysis

We performed a Western blot analysis as previously described [36]. Cells were washed twice with cold PBS, harvested, and then sonicated in lysis buffer containing 10 mM Tris-HCl buffer (pH 7.5), 1% SDS, 1% Triton X-100, and a protease inhibitor cocktail (Roche, Mannheim, Germany). The skin samples from mice were homogenized and sonicated in the lysis buffer. The protein concentration in each lysate was measured using a BCA protein assay kit (Pierce, IL, USA). Proteins in the supernatant were separated by electrophoresis on 10% SDS-polyacrylamide gels and transferred to a PVDF membrane. We detected each protein by incubation with each antibody followed by incubation with horseradish peroxidase-conjugated antibodies to IgG. Anti-IL-4 antibody was obtained from Bioworld (MN, USA). Anti-HMGB1 antibody and anti-α-SMA antibody were obtained from Genetex (CA, USA). Anti-Arg-1 antibody was obtained from Proteintech (IL, USA). Anti-α2AP antibody and anti-RAGE antibody were obtained from Santa Cruz Biotechnology (CA, USA). Anti-type I collagen antibody was obtained from Bioss (MA, USA). Anti-iNOS antibody was obtained from Anaspec (CA, USA). Anti-phospho-STAT6 antibody and anti-STAT6 antibody were obtained from Cusabio (TX, USA). Anti-GAPDH antibody was obtained from Sigma-Aldrich (MO, USA).

### Measurement of dermal thickness

The dermal thickness from the epidermal-dermal junction to dermal-subcutaneous junction was measured in skin sections, and it was determined by calculating the average of three-point measurement in each skin section. The measurements were carried out in a blinded fashion. The dermal thickness was measured in the skin sections from each group of mice.

### Sirius red staining

Sirius red staining was performed as previously described [37]. After deparaffinization, the skin sections were treated with 0.2% phosphomolybdic acid for 5 min. Next, the skin sections were stained with 0.1% sirius red for 90 min and 0.01 N HCl for 2 min.

### Immunohistochemical staining of α-SMA and CD206

Immunohistochemical staining of α-SMA and CD206 was performed as previously described [38]. Paraffin sections of mice were labeled with anti-α-SMA primary antibody (Genetex, CA, USA) or anti-CD206 primary antibody (R&D systems, MN, USA), then secondarily labeled with Cy3-conjugated anti-rabbit IgG or FITC-conjugated anti-goat IgG (Thermo Fisher Scientific, MA, USA), respectively. The signals were then detected using a laser-scanning microscope.

### siRNA study

We performed siRNA study as previously described [39, 40]. The dermal fibroblasts were transfected with RAGE siRNA (Santa Cruz Biotechnology, CA, USA) using Lipofectamine 2000 (Invitrogen, CA, USA) according to the manufacturer’s instructions. A non-specific siRNA was employed as the control. At 48 h after transfection, the cells were used for experiments.

### Statistical analysis

All data were expressed as mean ± SEM. The statistical analysis was conducted with unpaired *t* test for two-group comparison, with one-way ANOVA followed by the least significant difference test for multiple comparisons. Statistical significance was defined as a *P* value of < 0.05.

## Results

### HMGB1 induced pro-fibrotic changes and α2AP production through RAGE in fibroblasts

**Fig. 1:**
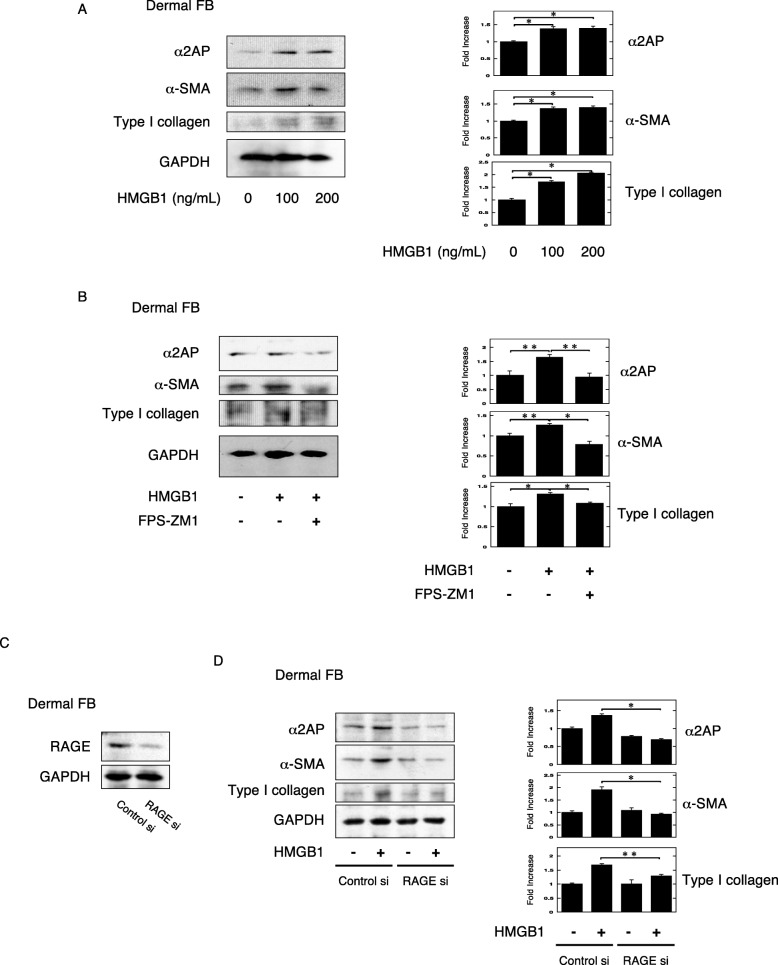
HMGB1 induced pro-fibrotic changes and α2AP production through RAGE in fibroblasts. **a** The dermal fibroblasts were stimulated by HMGB1 (100, 200 ng/ml) for 24 h. The expression of each protein was examined by a Western blot analysis. The histogram shows quantitative representations of each protein (*n* = 3). **b** The dermal fibroblasts were pretreated by FPS-ZM1 (100 μM) for 30 min and then stimulated by HMGB1 (200 ng/ml) for 24 h. The expression of each protein was examined by a Western blot analysis. **c** The dermal fibroblasts were transfected with control or RAGE siRNA. At 48 h after transfection, the cells were used for experiments. **d** The siRNA-transfected dermal fibroblasts were stimulated by HMGB1 (200 ng/ml) for 24 h. The expression of each protein was examined by a Western blot analysis. The histogram shows quantitative representations of each protein (*n* = 3). The data represent the mean ± SEM. *P < 0.01, **P < 0.05

First, we focused on HMGB1, which is associated with the development of fibrosis, and found that HMGB1 induced pro-fibrotic changes, such as an increase in the expression of α-smooth muscle actin (α-SMA) (a hallmark of the myofibroblast phenotype) and type I collagen, and α2AP production in the dermal fibroblasts (Fig. 1a). It has been reported that HMGB1 can bind to RAGE, and mediates fibroblast activity and myofibroblast differentiation [41, 42]. Therefore, we examined the effect of the RAGE-specific inhibitor FPS-ZM1 [43] on the HMGB1-induced pro-fibrotic effects and α2AP production in the dermal fibroblasts. FPS-ZM1 attenuated the HMGB1-induced pro-fibrotic changes and α2AP production (Fig. 1b). Furthermore, we investigated the effect of RAGE reduction in dermal fibroblasts by using siRNA (Fig. 1c). The reduction of RAGE markedly attenuated the HMGB1-induced pro-fibrotic changes and α2AP production (Fig. 1d). These data suggest that HMGB1 induced pro-fibrotic changes and α2AP production through RAGE in fibroblasts.

### The effects of immune cells on the development of fibrosis

**Fig. 2:**
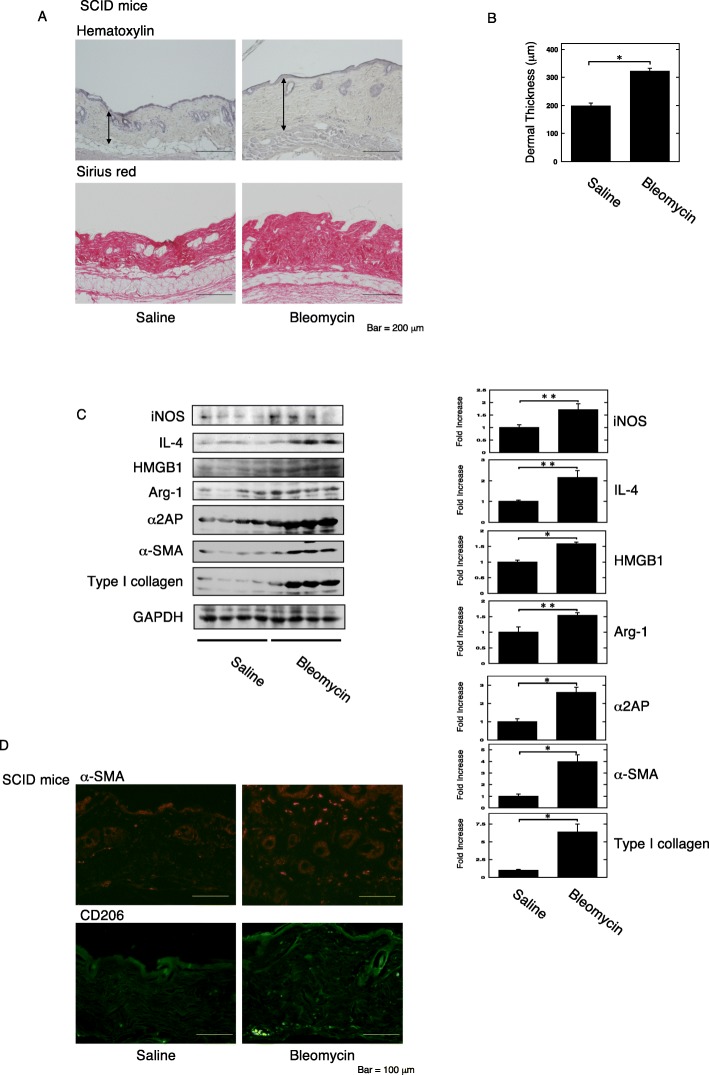
The effects of T and B cell-deficient severe combined immune deficiency in the bleomycin-treated mice. **a** Representative skin sections from saline- or bleomycin-administered SCID mice (hematoxylin and sirius red stain). Double head arrows indicate the dermal thickness. **b** The dermal thickness in the skin sections from mice (*n* = 4). **c** The expression of each protein in the skin from mice was examined by a Western blot analysis. The histogram shows quantitative representations of each protein (*n* = 4). **d** The skin sections of mice were stained with anti-α-SMA antibodies or anti-CD206 antibodies. The data represent the mean ± SEM. *P < 0.01, **P < 0.05

**Fig. 3:**
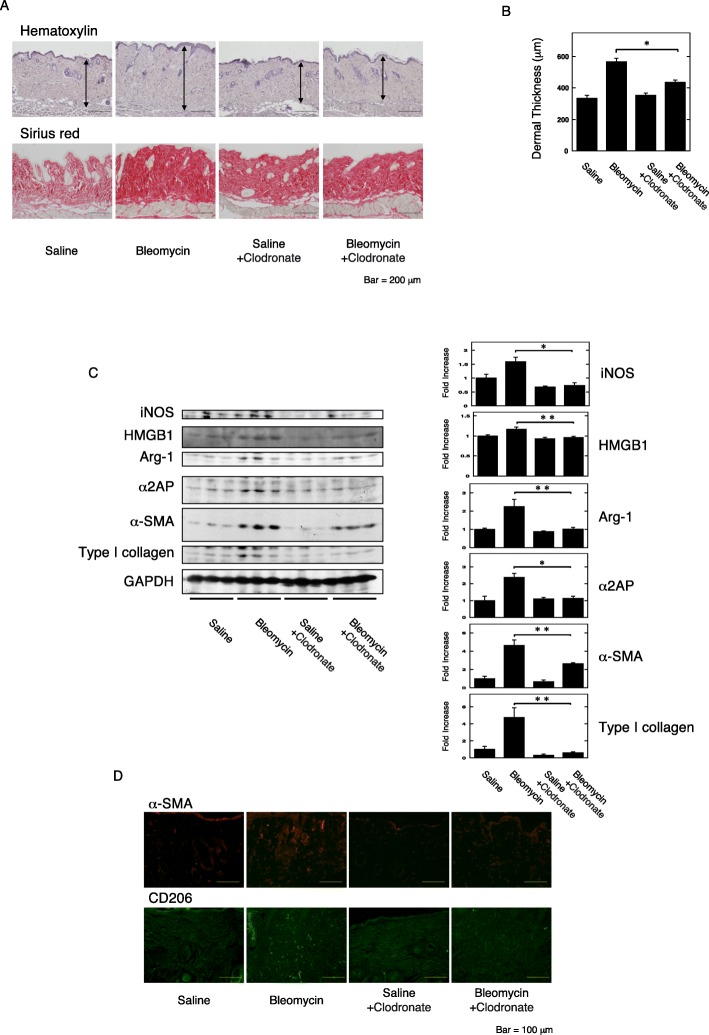
The effects of macrophage reduction in the bleomycin-treated mice. **a** Representative skin sections from saline-, bleomycin-, saline plus clodronate-, or bleomycin plus clodronate-administered mice (hematoxylin and sirius red stain). Double head arrows indicate the dermal thickness. **b** The dermal thickness in the skin sections from mice (*n* = 4). **c** The expression of each protein in the skin from mice was examined by a Western blot analysis. The histogram shows quantitative representations of each protein (*n* = 3). **d** The skin sections of mice were stained with anti-α-SMA antibodies or anti-CD206 antibodies. The data represent the mean ± SEM. *P < 0.01, **P < 0.05

HMGB1 is released from immune cells, such as T cells, B cells, macrophages, and the immune cells are associated with the development of fibrosis [4, 5]. Next, to clarify which immune cells are associated with the HMGB1 and α2AP production that occurs with the development of fibrosis, we examined the effects of T and B cells on belomycin-induced dermal fibrosis using T and B cell-deficient severe combined immune deficiency (SCID) mice. The administration of bleomycin in SCID mice induced pro-fibrotic changes, such as increased dermal thickness (Fig. 2a, b) and an increase in the expression of type I collagen, α-SMA, IL-4, α2AP, HMGB1, inducible nitric oxide synthase (iNOS) (a hallmark of the classically activated macrophage phenotype), and Arg-1 and CD206 (Arg-1 and CD206; a hallmark of the alternatively activated macrophage phenotype) in the skin (Fig. 2c, d). Furthermore, we examined the effects of macrophage reduction by clodronate [44] on the fibrosis progression and the HMGB1 and α2AP production. Macrophage reduction in the bleomycin-treated mice abolished the pro-fibrotic changes, such as increased dermal thickness (Fig. 3a, b) and an increase in the expression of type I collagen, α-SMA, α2AP, HMGB1, iNOS, and Arg-1 and CD206 in the skin (Fig. 3c, d). These data suggest that macrophages are associated with the HMGB1 and α2AP production that occurs with the process of fibrosis progression.

### The effects of alternatively activated macrophages on the progression of fibrosis and the production of α2AP and HMGB1

**Fig. 4:**
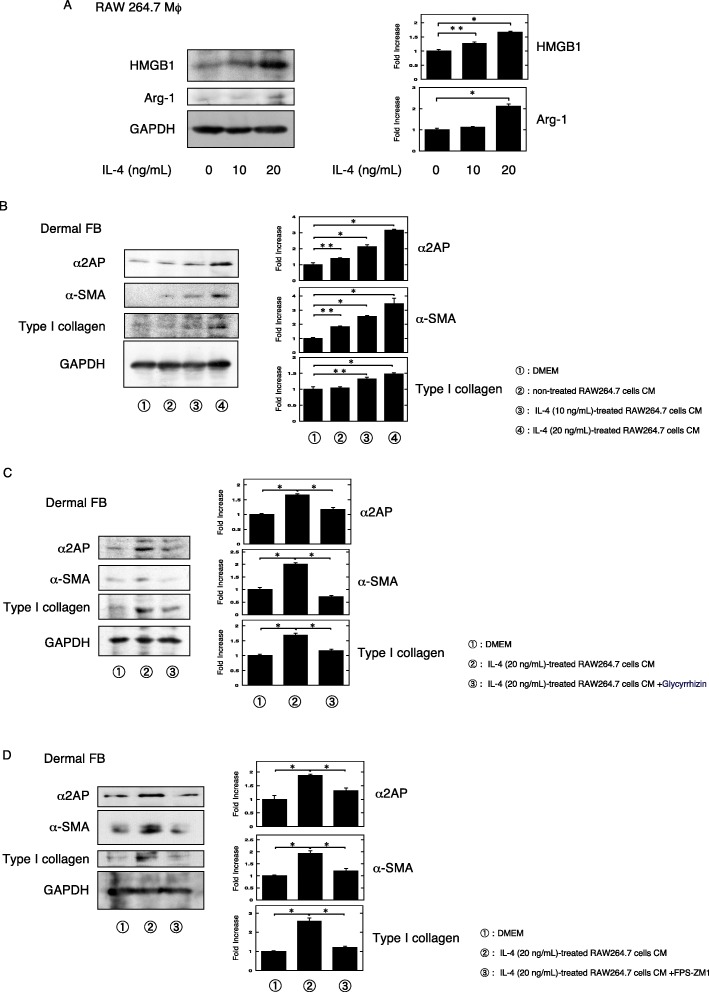
The effects of alternatively activated macrophages on the progression of fibrosis and the production of α2AP and HMGB1. **a** RAW 264.7 macrophages were stimulated by IL-4 (10, 20 ng/ml) for 48 h. The expression of each protein was examined by a Western blot analysis. The histogram shows quantitative representations of each protein (*n* = 3). **b** The dermal fibroblasts were cultured with the CM of the non-treated RAW 264.7 cells, the IL-4 (10 ng/ml)-treated RAW 264.7 cells, or the IL-4 (20 ng/ml)-treated RAW 264.7 cells for 24 h. The expression of each protein was examined by a Western blot analysis. The histogram shows quantitative representations of each protein (*n* = 3). **c** The dermal fibroblasts were cultured with the CM of the IL-4 (20 ng/ml)-treated RAW 264.7 cells supplemented with or without glycyrrhizin (100 μg/ml) for 24 h. The expression of each protein was examined by a Western blot analysis. The histogram shows quantitative representations of each protein (*n* = 3). **d** The dermal fibroblasts were treated with the CM of the IL-4 (20 ng/ml)-treated RAW 264.7 cells supplemented with or without FPS-ZM1 (200 μM) for 24 h. The expression of each protein was examined by a Western blot analysis. The histogram shows quantitative representations of each protein (*n* = 3). The data represent the mean ± SEM. *P < 0.01, **P < 0.05

Alternatively activated macrophages are associated with the development of dermal fibrosis [45, 46]. Thus, we examined the effects of alternatively activated macrophages on fibrosis progression and the production of HMGB1 and α2AP. Since IL-4 is known to induce alternatively activated macrophages, we then confirmed that IL-4 induced an increase in the Arg-1 expression in RAW 264.7 macrophages (Fig. 4a). Next, we examined the effects of alternatively activated macrophages on the development of fibrosis and showed that conditioned media (CM) of IL-4-treated RAW264.7 macrophages induced the pro-fibrotic changes, such as an increase in the expression of α-SMA, type I collagen, and α2AP production in the dermal fibroblasts (Fig. 4b). In addition, we examined the effect of the direct HMGB1 inhibitor glycyrrhizin [47] and showed that glycyrrhizin attenuated the IL-4-treated RAW 264.7 macrophage CM-induced pro-fibrotic changes and α2AP production in the dermal fibroblasts (Fig. 4c). Furthermore, we showed FPS-ZM1 attenuated the IL-4-treated RAW 264.7 macrophage CM-induced pro-fibrotic changes and α2AP production in the dermal fibroblasts (Fig. 4d). These data suggest that IL-4-stimulated alternatively activated macrophages produced HMGB1 and that the HMGB1 induced pro-fibrotic changes and α2AP production through RAGE in fibroblasts.

### The effects of IL-4Rα neutralization on the development of fibrosis

**Fig. 5:**
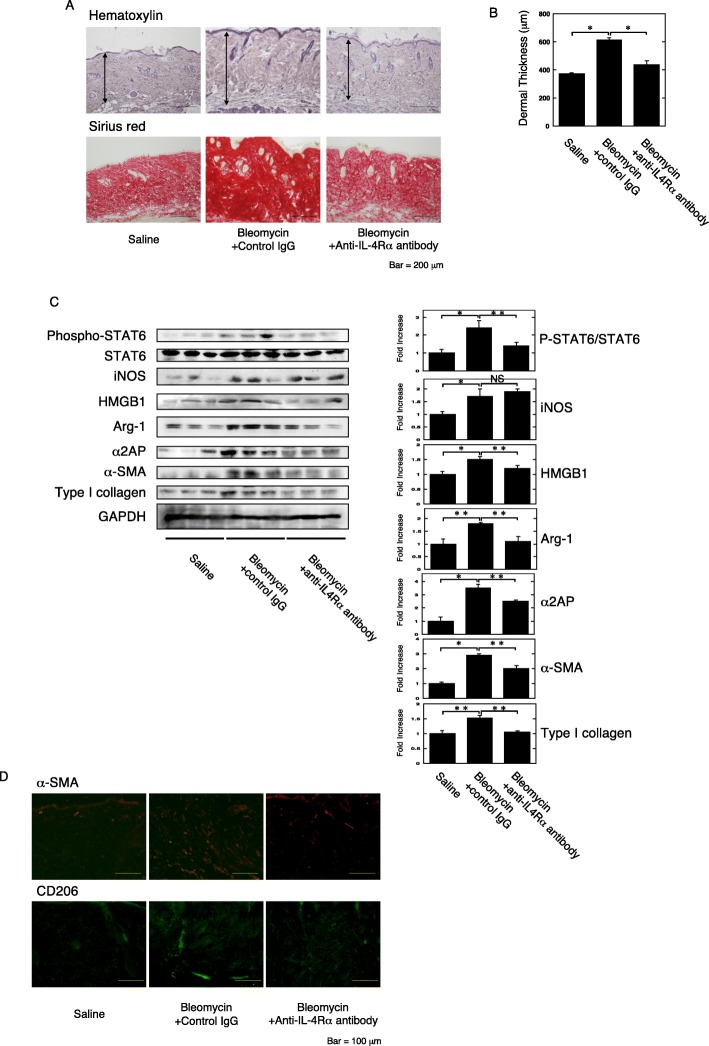
The effects of IL-4Rα neutralization in the bleomycin-treated mice. **a** Representative skin sections from saline-, bleomycin plus control IgG-, or bleomycin plus IL-4Rα neutralizing antibodies-administered mice (hematoxylin and sirius red stain). Double head arrows indicate the dermal thickness. **b** The dermal thickness in the skin sections from mice (*n* = 4). **c** The expression of each protein in the skin from mice was examined by a Western blot analysis. The histogram shows quantitative representations of each protein (*n* = 3). **d** The skin sections of mice were stained with anti-α-SMA antibodies or anti-CD206 antibodies. The data represent the mean ± SEM. *P < 0.01. **P < 0.05

Furthermore, to clarify whether IL-4 signaling is associated with the HMGB1 and α2AP production that occurs with the development of fibrosis, we examined the effects of IL-4 blocking by IL-4 receptor α (IL-4Rα) neutralization in bleomycin-treated mice. The administration of IL-4Rα neutralization antibodies to the bleomycin-treated mice attenuated the pro-fibrotic changes, such as increased dermal thickness (Fig. 5a, b) and an increase in the expression of type I collagen, α-SMA, α2AP, HMGB1, and Arg-1 and CD206 in the skin (Fig. 5c, d). In contrast, the IL-4Rα neutralization did not attenuate an increase in the expression of iNOS in the skin (Fig. 5c). Furthermore, we showed that the administration of IL-4Rα neutralization antibodies to the bleomycin-treated mice attenuated the STAT6 activation in the skin (Fig. 5c). These data suggest that IL-4 signaling is associated with the increase in alternatively activated macrophage numbers and the production of HMGB1 and α2AP that occurs with the process of fibrosis progression.

## Discussion

Fibrotic diseases are characterized by tissue overgrowth, hardening, and/or scarring due to the excessive production, deposition, and contraction of ECM. Recently, we showed that the expression of α2AP is elevated in fibrotic tissue and that the blockade of α2AP attenuates the development of fibrosis [11, 24, 25]. α2AP is associated with the production of pro-fibrotic factors, myofibroblast differentiation, ECM production, and plasmin inhibition [7, 11, 26], and increased α2AP may play an important role in the development of fibrosis. Thus, we examined the mechanism underlying the production of α2AP that occurs with the development of fibrosis.

In this study, we focused on HMGB1, which is known to contribute to inflammation, immune response, myofibroblast differentiation, ECM production, and progression of fibrosis [30–34], and found that HMGB1 induced pro-fibrotic changes and the production of α2AP through RAGE in fibroblasts (Fig. 1). In addition, we showed that macrophage reduction attenuated the pro-fibrotic changes and the HMGB1 and α2AP production that occurs with the process of fibrosis progression (Fig. 3). On the other hand, the pro-fibrotic changes and the HMGB1 and α2AP production were induced in bleomycin-treated T and B cell-deficient SCID mice (Fig. 2). Several studies have reported that T and B cells are not an essential requirement for the development of fibrosis [48, 49]. These data suggest that macrophages may play a pivotal role in the process of fibrosis progression and that macrophage-regulated production of HMGB1 and α2AP may be associated with the induction and development of fibrosis.

HMGB1 is secreted by activated macrophages and is associated with the polarization of classically and alternatively activated macrophage and the promotion of cytokine production in alternatively activated macrophage [50–53]. It has been reported that classically and alternatively activated macrophages are associated with the induction and development of inflammation and fibrosis [5, 54]. In particular, alternatively activated macrophages are elevated in SSc patients [55], and they are associated with the development of dermal fibrosis [45, 46]. In the present study, we showed that the expression of classically and alternatively activated macrophage markers was elevated in dermal fibrosis model mice. We also showed that the blockade of IL-4Rα attenuated the pro-fibrotic changes and the increase in the expression of α2AP, HMGB1, and alternatively activated macrophage markers in dermal fibrosis model mice, but not the increase in the expression of classically activated macrophage markers. These data suggest that IL-4 signaling is associated with the increase in alternatively activated macrophage numbers and the production of HMGB1 and α2AP that occurs with the process of fibrosis progression. In addition, HMGB1 may not affect the increase in classically activated macrophage numbers in dermal fibrosis model mice, and classically activated macrophages may be not an essential requirement for the IL-4-mediated fibrosis progression. Next, we showed that IL-4-stimulated alternatively activated macrophages produced HMGB1 (Fig. 4a) and that the IL-4-stimulated alternatively activated macrophage CM induced pro-fibrotic changes and the production of α2AP through HMGB1/RAGE in fibroblasts (Fig. 4). On the other hand, IL-4 did not induce the production of HMGB1 in fibroblasts (data not shown). These data suggest that IL-4-stimulated alternatively activated macrophages produced HMGB1, and the HMGB1 subsequently induced α2AP production in fibroblasts.

The serum level of IL-4 is elevated in bleomycin-treated mice [56, 57]. We showed that IL-4 was elevated in bleomycin-treated T and B cell-deficient SCID mice skin (Fig. 2c). IL-4 is mainly produced by T cells but is also produced by mast cells, basophils, and eosinophils [58]. These cells may be associated with the production of IL-4, and the increase of IL-4 may cause the induction and development of fibrosis. IL-4 is also known to regulate macrophage proliferation and accumulation, and to induce collagen synthesis and fibroblast proliferation, and is associated with the development of fibrosis [59–62]. Furthermore, we showed that the blockade of IL-4 signaling attenuated the pro-fibrotic changes, the increase in alternatively activated macrophage numbers, and the production of HMGB1 and α2AP that occurred with the process of fibrosis progression (Fig. 5). In addition, the IL-4Rα signaling regulates IL-4-induced STAT6 activation [63]. We showed that the blockade of IL-4Rα attenuated the STAT6 activation in dermal fibrosis model mice. It has been reported that the inhibition of STAT6 causes the resolution of lung inflammation and fibrosis in the bleomycin-treated mice [64], and the STAT6 pathway is associated with bone marrow-derived fibroblast activation [65]. The attenuation of STAT6 by IL-4Rα neutralizing may contribute to the development of dermal fibrosis. These data suggest that the IL-4 signaling plays an important role on the induction and development of fibrosis, and the blockade of IL-4 signaling may be a potential target for novel therapies that prevent fibrotic diseases.

## Conclusion

Alternatively activated macrophage-derived HMGB1 induced the α2AP production through RAGE, and these effects are associated with the development of fibrosis. Our findings may provide a novel therapeutic approach to the treatment of fibrotic disorder.

## Data Availability

The authors declare that all data supporting the findings of this study are available within the article.

## Abbreviations

α2AP: α2-Antiplasmin
α-SMA: α-Smooth muscle actin
ATGL: Adipose triglyceride lipase
CM: Conditioned media
DMEM: Dulbecco’s modified Eagle’s medium
ECM: Extracellular matrix
FCS: Fetal calf serum
HGF: Hepatocyte growth factor
HMGB1: High-mobility group box 1
IL-4Rα: IL-4 receptor α
iNOS: Inducible nitric oxide synthase
iPLA_2_: Calcium-independent phospholipase A_2_
MMPs: Metalloproteinases
PAP: Plasmin-α2AP
PNPLA2: Patatin-like phospholipase domain-contain 2
RAGE: Receptor for advanced glycation end products
SCID: Severe combined immune deficiency
SSc: Systemic sclerosis
TGF-β: Transforming growth factor-β
